# Updates on the Latest Surgical Approach of the Aortic Stenosis

**DOI:** 10.3390/jcm10215140

**Published:** 2021-10-31

**Authors:** Lucian Geicu, Olivier Busuttil, Nicolas D’Ostrevy, Mathieu Pernot, Walid Benali, Louis Labrousse, Thomas Modine

**Affiliations:** 1 Department of Cardiac Surgery, Montreal Heart Institute, Montreal University, Montreal, QC H1T 1C8, Canada; geicu.luc@gmail.com (L.G.); dr.walidbenali@gmail.com (W.B.); 2 Unité Médico-Chirurgicale, Hôpital Haut Lévêque, CHU de Bordeaux, CEDEX, 33604 Pessac, France; olivierbusuttil@chu-bordeaux.fr (O.B.); mathieupernot33@gmail.com (M.P.); louis.labrousse@chu-bordeaux.fr (L.L.); 3 Cardiac Surgery Department, CHU de Clermont Ferrand, 63003 Clermont Ferrand, France; ndostrevy@chu-clermontferrand.fr

**Keywords:** AVR, new valve technologies, mini-invasive surgery, Ross procedure, aortic root  enlargement, TAVI, V-in-V, decision making algorithm

## Abstract

Over the last twenty years, we marked significant progresses in the field of tissue engineering and the development of new aortic valve structural and delivery systems. These continuous iterations on the field, have completely changed the surgical indications and approaches for AVR. Nowadays, therapeutic decisions are endorsed by international guidelines; however, new technical advances need a new integrated approach. The clinical scenarios issued from the interaction between the Guidelines and the newest approaches and technologies are regularly on debate by the Heart Team. We will present some of our most encountered situations and the pattern of our therapeutic decisions. To easily navigate through Guidelines and clinical scenarios, we reported in this review a simplified and easy to use Clinical decision-making algorithm that may be a valuable tool in our daily practice.

## 1. Introduction

In 1957, Walton Lillehei performed the first intracardiac aortic valve replacement with a plastic prosthesis. In 1960, in Boston, Harken replaced a stenosed aortic valve below the level of the coronary ostia with a caged-ball prosthesis [1].

Over the last twenty years, we marked significates progresses in the field of tissue engineering and the development of new aortic valve structural and delivery systems. These continuous iterations on the field, have completely changed the surgical indications and approaches for AVR.

Nowadays, therapeutic decisions are endorsed by international guidelines, however new technical advances need a new integrated approach.

As with any transition, the process seems to be long and the community needs to be constantly informed with regard to new available technologies, techniques and approach strategies.

In the present review, we will focus on (1) updates on the latest surgical approaches and techniques and (2) decision making algorithm to help practitioners to choose the optimal technique and approach for their patient.

### 1.1. Historical Background and Recent Considerations

For many years, a full sternotomy was the only way to get access to the aortic valve and it is still considered to be the golden standard. For SAVR, there are the two traditional substitutes: the biological and the mechanical prosthesis. Due to our special interest in the matter, we shall take a closer look at the newest generation of valves including the rapid deployment valves.

New technologies have brought significant advancement, achieved the same safety and efficacy of traditional SAVR and offered less invasive procedures. Top of the list are the TAVI/TAVR procedures with all their possible accesses. Some groups have argued that this is the only truly minimally invasive approach for aortic valve stenotic pathologies; however, from our point of view, the truth seems to lie somewhere in the middle. Although initial clinical results are satisfactory, to date, we have only eight years with TAVI [2] compared to longer follow-up with surgery [3,4]. TAVI is a valuable procedure with a multitude of benefits for an increasing category of patients and now validated across all surgical profiles [5,6]. Newly emerged data from expert centers require a refocusing on the Ross procedure, the potential benefits of this procedure and its place in the surgical tool box will be discussed in this review. Considering the possibility of V-in-V procedures, we will expose the potential long-term advantages of root enlargement during the initial surgery (Nicks, Nunez, Manouguian, Kono-Rastan) [7] and a new technique recently described: the Y incision with rectangular patch to enlarge the aortic annulus [8,9].

The key question is how to treat patients with aortic stenosis in the perspective of the best immediate and long-term results, ensuring good quality of life and low combined operative risk. The solution may reside in patient selection, and the goal of the Heart Teams is to identify the best durable strategy considering the patient’s life expectancy and desire.

### 1.2. Updates on the Latest Surgical Approaches and Techniques

Conventional surgical aortic valve replacement has already been described in numerous scientific articles; therefore, we choose to focus on the “newest” approaches.

#### 1.2.1. Minimally Invasive Aortic Valve Surgery

In the late 1990s, various groups started reporting about minimally invasive surgery with encouraging results. The procedure was facilitated by technological advances in the industry. We witnessed the emergence of new types of valves, adapted instruments, better optics, and creation of proctoring programs.

Ueno t al. [10] sum up some of the techniques that could be used. They mention full sternotomy with limited skin incision. The technique was initially described by Luciani et al. [11]. This approach may be perceived as a transition towards smaller incision, offering the security of midline access with the possibility of rapid conversion. The right parasternal approach, a procedure which eventually evolved into what we presently refer to as mini-sternotomies was also cited. This approach allows surgeons to use conventional techniques through a smaller incision, while simultaneously ensuring a transition to thoracotomies.

In recent years, different levels of minimally invasive surgery have emerged, with progression in minimizing the approach, first from a minimal skin incision with complete sternotomy, to a mini-sternotomy. The minimization was pushed farther to the first mini-thoracotomy, where there is no longer any sternal involvement. Technical advances enabled the development from mini-thoracotomy with costal cartilage section, towards even less traumatic mini-thoracotomy, which allows for complete preservation of costochondral integrity.

Three different types of approaches are reported in the literature: the Right Anterior Mini- Thoracotomy (RAMT [12]), the Right Anterolateral Mini-Thoracotomy (RALT [13]) and the Right Vertical Infra-Axillary Thoracotomy, described by several research groups from Asia [14,15,16].

The “J” partial mini-sternotomy and the right anterior mini thoracotomy were identified as the two main methods for minimally invasive AVR.

Literature in the field still evinces a confusion between minimally invasive surgery, including mini-sternotomy, and mini-thoracotomy; however, the concepts were already described and clarified by Glauber in 2015 [17]. The article became a state-of-the-art point of reference, proving that minimally invasive approaches consist of any surgery performed by approaches other than a total sternotomy. Glauber makes it clear that the majority of studies showcasing the value of minimally invasive approaches were based on mini-sternotomy. This first minimally invasive approach demonstrated a reduction in mortality, morbidity, postoperative pain and transfusion. The mini-thoracotomy approach was then developed by their team starting with 2005, in light of advances in the field of mini-sternotomy surgery. Severely obese patients are more likely to benefit from this procedure [18]. Prolonged operating, clamping and pump times, therefore represent the major drawbacks of this technique.

In what mini-thoracotomy is concerned, it has already been demonstrated that it leads to a reduction in complications, infections, transfusions, bleeding, supraventricular rhythm disturbances, but also to a reduction in the length of Intensive Care unit and hospital stays [19].

However, in light of Glauber's experience, legitimate questions arise regarding the ease and especially the difficulties of switching from simple sternotomy surgery to a more complex surgery performed by mini-thoracotomy. Several groups have advanced the idea of clinical and radiological [20,21] criteria, to be considered prior to surgery, which would make it possible to assess the pros and cons of performing a mini-thoracotomy.

Without being extensive, Klein [22] and more recently Ramchadani [12] reported their exclusion criteria for minimally invasive approaches. Patients who underwent high dose chest radiation, who evince morphological chest wall deformities, underwent previous lung surgeries and evince any other pathologies that may change the position of the mediastinum, should be directed towards other possible approaches. Severely atherosclerotic aorta and vessels is also an absolute contraindication. In case of previous heart surgery without CABG or patients without history of pericardial pathology, we may take into consideration mini-thoracotomy.

If TAVI has been consistently compared to sternotomy surgery, what about comparing it also to minimally invasive approaches? Pahn’s meta-analysis, showcased similar results in the case of sternotomy, mini-sternotomy and mini-thoracotomy, although clamping times are extended during mini-thoracotomy procedures [23].

The occurrence of strokes has always been an important issue in aortic valve replacement surgery. At a time when the point of comparison is no longer surgery by sternotomy, but aortic replacement by TAVI, retrograde extracorporeal circulation leads to a probable increase in risk. Patients with abdominal atheroma are probably most at risk for a brain event. Radiological stroke rates of around 1.5% are mentioned by the literature [24]. In this kind of patient mini-sternotomy allowing anterograde CPB can be the best option.

#### 1.2.2. “New” Materials: Sutureless Prosthesis

Advances in technique and innovation facilitated the development of new rapid deployment and sutureless valves. Two of these have passed the test of time and are largely used worldwide. Intuity (Edwards Lifesciences, Irvine, CA, USA) is commonly described as a “rapid deployment” valve, whereas the Perceval valve (LivaNova, London, United Kingdom) is generally referred as a “sutureless valve”. Preliminary experience dates back to 2008.

Fischlein’s study [25] revealed that sutureless valves were non inferior to stented valves with respect to major adverse cerebral and cardiovascular events after one year in patients undergoing aortic valve replacement (alone or with coronary artery bypass grafting). Aortic valve hemodynamics improved equally in both groups, the incidence of paravalvular leaks was similar, whereas there was a higher rate of pacemaker implantation in the sutureless group after one year (11.1% vs 3.6%).

In a recent meta-analysis of postoperative and mid-term results in isolated aortic valve replacement [26], six comparative studies focusing on the Perceval aortic valve and conventional stented bioprostheses were identified. There was no difference in one year mortality. The incidence of renal failure and blood transfusion was lower in the Perceval group. The incidence of paravalvular leaks and strokes was similar, even if paravalvular leaks were on average double (3.1% vs. 1.6%) in the Perceval group. The Perceval group had significantly higher rates of pacemaker implantation, whereas the performance of the prosthesis was better in term of mean gradient at 1 year of follow up. The difference is statistically significant but may not be clinically significant.

Up to five years of outcomes on 1998 patients receiving a Perceval or Intuity valve, were reported by Williams et al. [27]. Inclusion criteria for this systematic review included survival and postoperative echocardiographic outcomes greater than five years. A total of42.4% were operated by full sternotomy, 31.6% for mini-sternotomy and 24.9% of patients underwent right anterior thoracotomy. Overall survival rates at the five-year of follow-up were 84.2%. Incidence of strokes (4.8%), severe paravalvular leaks (1.5%), and permanent pacemaker insertion (8.2%) at up to five-year follow-up were acceptable for the authors. Hemodynamic outcomes were also found acceptable for the authors (mean pressure gradient 8.8–13.6 mmHg, peak pressure gradient 18.9–21.1 mmHg) and effective orifice area (EOA) (1.5–1.8 cm^2^).

PERSIST AVR [28] is the first prospective, randomized study comparing in-hospital and post-discharge outcomes in a robust population of patients undergoing SAVR with either the Perceval sutureless bioprosthesis or a conventional sutured stented bio-prosthesis up to 5 years. A total of 910 patients were randomized in 47 centers. The preliminary results are waited for 2023. Sutureless prostheses may be of particular interest for complex and combined procedures and to a lesser degree for small aortic annulus and heavily calcified aortic roots [29].

Other series affirm that sutureless prostheses perform well in small aortic annulus [30,31,32]. However, Ghoneim et al., in a single center retrospective study which compares stented, stentless, sutureless, and root enlargement patients, showed that stentless bio-prostheses and Trifecta stented valves had the best hemodynamic outcomes [33].

Finally, sutureless bio-prostheses could be a good alternative particularly in long time combined procedures, minimally invasive aortic surgery, heavily calcified aortic root and to a lesser degree, in small aortic annulus.

#### 1.2.3. The Ross Procedure

For many years, the two main options for young adults were primary mechanical valves and as backup option, the bio-prosthesis. Considering all inconveniences of mechanical valves, the use of bio-prostheses took over patient preferences and has consistently increased over the last 10 years [34,35]. Hoping for a better quality of life and being well informed with regard to new available technologies, patients are now tempted to accept a second and even a third operation in case of SVD. In order to decrease all possible inconveniences of the prosthesis, particularly the mechanical ones, and subsequent redo’s, we hold that maybe “Is it Time to Reconsider Use of the Ross Procedure for Adults?” [36]. After an initial peak of enthusiasm in the early 1990s, the surgical community lost interest in the Ross procedure. Lack of data, surgical complexity, and the idea that it replaces a single valve disease with a double valse disease [37], halted the wide use of this type of surgery.

However, the Ross procedure had its partisans who continuously reported their clinical experiences and outcomes. Nowadays we can rely on a well-standardized procedure supported by a satisfactory number of studies. As Chauvette et al. mentioned recently, the known benefits are now combined with lessons learned from past experiences and risk mitigation strategies [38].

The positive results of the Ross procedure are strongly connected with patient selection and rigorous surgical technique. The hemodynamics and biological features of the pulmonary valve are key components of these equations. The superiority of the pulmonary autograft over all other protheses was endorsed by clinical, histological and radiological exams.

In spite of cumulative evidence showing good results associated with the Ross procedure, practice guidelines of the European society omit to mention Ross as surgical option or place it as a class IIb recommendation (AHA/ACC) [5,39].

In a propensity matched cohort study, Mazine et al. found long-term survival and freedom from reintervention were comparable between the Ross procedure and mechanical AVR. However, the Ross procedure was associated with improved freedom from cardiac and valve related mortality and a significant reduction in the incidence of stroke and major bleeding [40]. Furthermore, quality of life was better with pulmonary autografts [41].

In a recent study, Tam et al. showed that life expectancy was improved with the Ross procedure when the perioperative mortality rate was <2.5% and was equivalent to mechanical AVR when the mortality rate was 2.5% to 5% [42].

For David et al. and Sievers et al., 20 years freedom from reintervention on the autograft and the homograft exceed 90% and the operative mortality was less than 0.5% [43,44].

Furthermore, TAVI is possible in case of autograft and homograft failure [45,46]. Elsewhere pulmonary autograft inclusion in Dacron is probably the best practice to prepare TAVR procedure and avoid reintervention for root aneurysm [47].

Chauvette et al. synthesize the main results of contemporary studies reporting long-term outcomes. Freedom from reintervention after 15 years ranges from 75% to 94%, i.e., with a cumulative risk of 1–1.5%/patient-year and the survival rate was found to be between 89% and 99% [38].

The Algorithm for Patient Selection for the Ross procedure was found to be the same in expert centers. The ideal patient is a young/middle-aged adult with unrepairable valve disease; high levels of physical activity or women contemplating pregnancy. The ideal anatomic substrate is the aortic stenosis and normal-sized aortic annulus [48]. Patients with familial aortopathy, connective tissue disorder, autoimmune disorder or limited life expectancy (<15 years) are excluded. Suboptimal anatomic substrate is the aortic insufficiency or mixed lesions with predominant aortic insufficiency; dilatated annulus (>27 mm) and aortic/pulmonary size (mismatch >2 mm). In this case, a modified Ross procedure with autograft reinforcement with Dacron inclusion may be the solution [49]. Tight postoperative blood pressure control with beta-blockers in the range of 110–115 mmHg is recommended for 6 to 12 months [38,39,48].

Recently, the operative technique was described step-by-step by the group of Mount Sinai, NY, USA [39,50].

First, the autograft implantation can be performed using two different techniques: the sub-coronary, originally described by Donald Ross [51] and the root replacement technique. Because of the annulus size discrepancy, particularly in patients with aortic insufficiency or bicuspid aortic valve, the initial sub-coronary technique can be technically challenging. As a result, numerous teams have shifted to a full root replacement technique. While simplifying implantation, this technique exposes the pulmonary root to systemic pressures, frequently resulting in dilatation [52]. To avoid this complication, numerous technical modifications have been described.

As a first solution, the pulmonary autograft can be included in the native aortic root [53,54]. Reinforcement of the autograft with a prosthetic Dacron has been proposed recently to prevent late dilation of the autograft [55]. Another frequent mechanism of failure is the annular dilation leading to autograft insufficiency. Systematic performance of an extra-aortic annuloplasty with a circular Dacron ring has been suggested but longer follow up is needed.

Finally, to prevent sino-tubular dilatation and stabilize the segment, it is imperative to cut the autograft right above the commissure, and in case of slight aortic dilatation, do not hesitate to replace a segment of the ascending aorta. For the Montreal group, the cutoff for interposing a short Dacron graft is an aortic diameter of >35–40 mm depending on body habitus [38]. Finally, all the technical elements have the unique objective of stabilizing the pulmonary autograft at its different levels: annulus, sinus of Valsalva, and sino-tubular junction; as well as mitigating the risk of pulmonary homograft dysfunction.

The excellent long-term hemodynamics and low rates of valve related complications make this procedure a valuable tool which significantly benefits young patients.

#### 1.2.4. Aortic Root Enlargement

During the last decade, the use of biological valves increased substantially. With the perspective of V-in-V procedures gaining weight in our therapeutic decisions, surgeons must consider the possibility of an aortic root enlargement to avoid patient prosthesis mismatch (PPM). Indeed, in a meta-analysis Stuart et al. showed that PPM is associated with an increase in all cause and cardiac related mortality over long term follow up [56]. Worse outcomes of V-in-V TAVR have been reported in patients with small (<21 mm) or intermediate (21–25 mm) sized bioprostheses [57,58].

PPM in young adults results in a decline in predicted exercise capacity, delayed regression of left ventricular hypertrophy, a higher rate of structural valve degeneration and higher mortality [59,60]. Pre-existing PPM of the failed surgical valve is strongly and independently associated with increased risk for mortality following V-in-V implantation [61] and actually should contraindicate the procedure.

It is thus obvious that preparation for another possible surgery should start during the initial surgery. For optimal valve performance at rest and exercise, the indexed EOA at rest should ideally be no less than 0.85 to 0.90 cm^2^/m^2^ [59].

In the probably largest analysis to date (a recent propensity score analysis from a single center study) aortic root enlargement was not associated with increased risk of mortality or adverse events [57]. The results of the article must however be mitigated, two techniques were used (root enlargement and annulus plus root enlargement). It is likely that the morbidity and mortality is different depending on the technique, particularly in the Kono procedure which can be complex.

First described by Rahimtoola [62] in 1978, the technique was improved and, nowadays, we can resort to three posterior aortic root enlargement techniques (Manouguian, Nick and Nunez), as well as an anterior one, most frequently used in pediatric cardiac surgery (Konno-Rastan) [7]. In addition to these sometimes-old procedures, there is a recently published technique of rectangular patching after Y-cutting of the aortic root [8,9]. With the help of those techniques, it is possible to implant a valve 2 sizes larger than what the native annulus would normally accept. Bearing in mind the aforementioned results, mastering the techniques of aortic root enlargement is a “must” in our daily activity as they may improve immediate result and by permitting valve in valve procedures. 

### 1.3. Updates on the Management of the Latest Surgical Approaches: Decision-Making Algorithm in Daily Practice

When aortic valve replacement is necessary, one must keep in mind and evaluate: the valve’s hemodynamical profile, thrombogenesis, as well as resistance to infections, mode and rate of degeneration. Clinical characteristics of the patient are of paramount importance for our decision; life expectancy, contraindication for anticoagulation or treatment surveillance capacity, physical activity, anatomical particularities, but also expertise and access to resources and health services are to be taken in consideration. All of the above-mentioned factors contribute to long-term results. The aim is to have the best “global “survival which integrated the cumulative risk of procedures (including risk of reintervention) with the best quality of life.

**Figure 1:**
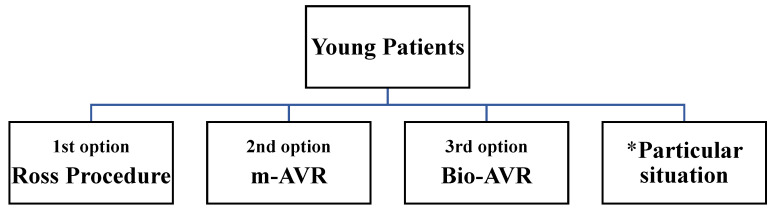
Priority of aortic valve substitutes for young patients.

**Figure 2:**
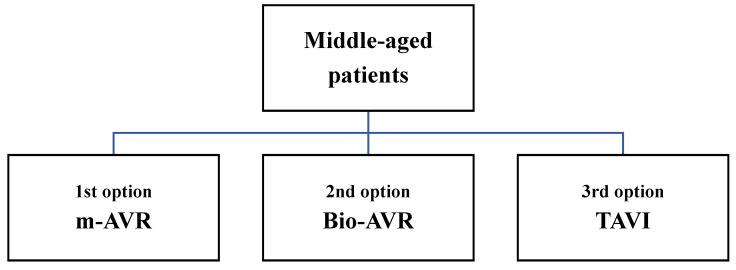
Priority of aortic valve substitutes for middle-aged patients.

**Figure 3:**
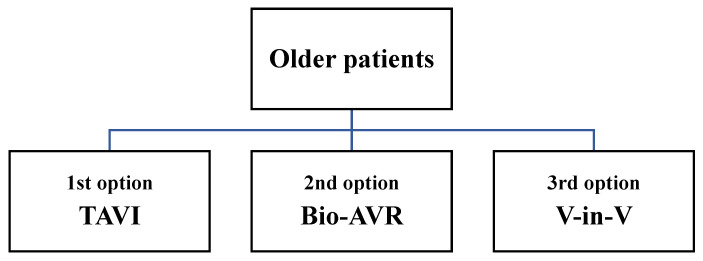
Priority of aortic valve substitutes for older patients.

We present the valves substitutes in order of our preferences related to the age (Figure 1Figure 2 and Figure 3) and life expectancy, with advantages and possible disadvantages.

Thus, the choice of valve substitute is a multidisciplinary decision (surgeon, cardiologist, anesthesiologist, and often geriatrician) adapted to the patient’s characteristics, alongside their values and preferences. To facilitate the daily practice, we tried to conceived a comprehensive and easy to use Decision Making Algorithm for choosing the right type of prothesis (Table 1, Table 2, Table 3, Table 4, Table 5, Table 6, Table 7, Table 8 and Table 9).

**Table 1:** Decision Making Algorithm. Ross Procedure for young patients.

Not Mentioned in the EACTS/ESC Guidelines from 2021 [6]	Supported by ACC/AHA Guidelines from 2020 [5] -Class 2b for Patients Younger than 50 Years
Pros	Cons
- Increasing level of evidence - Low rate of valve related complications - Possibilities of percutaneous procedure for valves degenerescence - Better quality of life - Hemodynamic superiority - No need for anticoagulation - Excellent choice for actives persons - Proved long time survival - Feasible by full sternotomy with reduced skin incision	- Still need to be supported by strong evidence data - Complex procedure; need to be done in high volume aortic centers - Only few mentorships’ programs and long learning curve - Low availability of pulmonic homograft’s - Complex redo procedure - Costly operation - Bad reputation to rehabilitate

**Table 2:** Decision Making Algorithm. m-AVR for young patients.

Supported by EACTS/ESC Guidelines from 2021 [6]	Supported by ACC/AHA Guidelines from 2020 [5]
- Class I SAVR for patients with STS-PROM/EuroSCORE II < 4%, who ask for this type of valve, high risk for accelerated SVD or no contraindication for anticoagulation, - Class IIa for patient already anticoagulated for another mechanical valve, for <60 years. or at high risks of reoperation or TAVI (if appropriate) - Class IIb for patient on long-term anticoagulation.	- Class 1 SAVR for symptomatic, well-informed patients who ask for this type of valve, no contraindication for anticoagulation, >20 years of life expectancy, - Class 1 SAVR for asymptomatic patients, <50% LVEF, >20 years life expectancy or rapid progression\urgency (abnormal exercise test, elevated BNP) - Class 2a for patients younger than < 50 years
**Pros**	**Cons**
- Good level of evidence - Long-term functionality - Easy to train - Improved hemodynamics and thrombogenicity - Theoretically, no need of reoperation - Alternatives accesses to total sternotomies, available - Aortic root enlargement possible - Patient already on anticoagulation for life	- Lifelong anticoagulation - Rigorous control of anticoagulation are needed - Restriction in day-by-day activities (recreational or professional) - Psychological disturbances and intellectual level that does not guarantee satisfactory compliance - Noisy for underweighted patients - Hemorrhagically or thrombotic risks dues to age acquired pathologies (loos of memory, tumoral pathologies, increased risks for socially neglected patients) - Not recommended for women contemplating pregnancy

**Table 3:** Decision Making Algorithm. Bio-SAVR for young patients.

Supported by EACTS/ESC Guidelines from 2021 [6]	Supported by ACC/AHA Guidelines from 2020 [5]
- Class I SAVR for: patient desire, risks for anticoagulation, life expectancy lower than the presumed durability of the prothesis, previous mechanical valve thrombosis or STS-PROM/EuroSCORE II < 4% - Class IIa for low likelihood or low risks for future redo or women contemplating pregnancy. - Class IIb for patients on long-term NOAC for high risk of thromboembolism	- Class 1 SAVR for symptomatic or asymptomatic well-informed patients of any age who ask for this type of valve, with contraindication for anticoagulation, with or without depressed LVEF (<50%), >20 years of life expectancy, rapid progression or urgency (abnormal exercise test, elevated BNP) - Class 2b percutaneous balloon dilatation as bridge to SAVR or TAVI for critically ill patient
**Pros**	**Cons**
- Good level of evidence; good long-term data - Promising new available technologies incorporated into the recent generations - Easy to use; early mentored by the residency programs - Multiple’s approaches/access available - Good choice in case of complex or urgent surgeries - Good choice for women contemplating pregnancy	- SVD in this category of age with increased risk of reoperation
- Quality of life over the mechanical valves - An option for anticoagulation contraindication - Annular enlargement possible - Valve-in-Valve may be possible in large prothesis. - Good choice for patients with different types of handicaps	

**Table 4:** Decision Making Algorithm. m-AVR for middle-aged patients.

Supported by EACTS/ESC Guidelines from 2021 [6]	Supported by ACC/AHA Guidelines from 2020 [5]
- Class I SAVR for patients with STS-PROM/EuroSCORE II <4 %, who ask for this type of valve, no contraindication for anticoagulation or high risk for accelerated SVD - Class IIa for patient already anticoagulated for another mechanical valve, for <60 years, high risks of reoperation or TAVI (if appropriate) - Class IIb for patient on long-term anticoagulation.	- Class 1 SAVR for symptomatic, well-informed patients who ask for this type of valve, no contraindication for anticoagulation, >20 years of life expectancy, - Class 1 SAVR for asymptomatic patients, <50% LVEF, >20 years life expectancy or rapid progression\urgency (abnormal exercise test, elevated BNP) - Class 2a for patients younger than <50 years - Class 2a for patients 50–65 years, informed shared decision, depending on patient characteristics
**Pros**	**Cons**
- All previously mentioned benefits for mAVR - available option for well-informed patients, compliant to the treatment and socially supported - May be “once in a lifetime procedure” in this category of age	- all the previously mentioned risks for a mechanical valve in young population.
- Good option for patients who experience fear of any other intervention	

**Table 5:** Decision Making Algorithm. Bio-SAVR for middle-aged patients.

Supported by EACTS/ESC Guidelines from 2021 [6]	Supported by ACC/AHA Guidelines from 2020 [5]
- Class I SAVR for: patient desire, risks for anticoagulation, life expectancy lower than the presumed durability of the prothesis, previous mechanical valve thrombosis, <75 years., STS- PROM/EuroSCORE II < 4% and operable but unsuitable for TAVI - Class IIa for low likelihood, low risks for future redo or aged >65 years - Class IIb for patients already on NOAC for high risks of thromboembolism.	- Class 1 SAVR for symptomatic or asymptomatic well-informed patients of any age who ask for this type of valve, with contraindication for anticoagulation, with or without depressed LVEF (<50%), >20 years of life expectancy, rapid progression or urgency (abnormal exercise test, elevated BNP) - Class 2a for 50–65 years -shared decision with accent on patient individualized treatment for the type of valve (biological or mechanical) - Class 2b percutaneous balloon dilatation as bridge to SAVR or TAVI for critically ill patient
**Pros**	**Cons**
- All previously mentioned advantages for AVR in young patients - Redo’s SAVR are possible in case of SVD - If certain criteria are presents, TAVI may be seen as an alternative to redo’s surgeries - Aortic root enlargement is possible in order to prepare for a later TAVI	- Risks of SVD still present

**Table 6:** Decision Making Algorithm. TAVI for middle-aged patients.

Supported by EACTS/ESC Guidelines from 2021 [6]	Supported by ACC/AHA Guidelines from 2020 [5]
- Class I for patients at high risks (STS-PROM/EuroSCORE II > 8%) and unsuitable for surgery - Class IIb–non-transfemoral TAVI may be considered for inoperable or unsuitable for TF- TAVI and balloon dilatation may be seen as a bridge to SAVR or TAVI	- Class 1 of recommendation for high prohibitive surgical risk with >1 year of life expectancy - Class 2b percutaneous balloon dilatation as bridge to SAVR or TAVI for critically ill patient
**Pros**	**Cons**
- An alternative for patients who refuse more invasive procedures although the choice of technique is that of the heart team and not of the patient - Multiples approaches available	- not supported by long term results high risk of permanent pace maker

**Table 7:** Decision Making Algorithm. TAVI for older patients.

Supported by EACTS/ESC Guidelines from 2021 [6]	Supported by ACC/AHA Guidelines from 2020 [5]
- Class I for patients at high risks (STS-PROM/EuroSCORE II > 8%), unsuitable for surgery and ≥75 years - Class IIb non-trans femoral TAVI may be considered for inoperable or unsuitable for TF- TAVI and balloon dilatation may be seen as a bridge to SAVR or TAVI	- Class 1 for symptomatic 65–80 years: shared decision for the type of approach: SAVR or TF- TAVI - Class 1 for symptomatic octogenarians with life expectancy <10 years with or without depressed LVEF (<50%) - Class 1 for any age with surgical prohibitive risks and life expectancy >12 months - Class 2b for critically ill patients, balloon dilatation may be considered as a bridge to SAVR or TAVI.
**Pros**	**Cons**
- Less invasive procedure - Option for patients already operated - In some cases of chest irradiation, porcelain aorta or chest deformation - Multiples access possible	- Lack of long-term data - Higher risk of AVC and paravalvular leak - Infective endocarditis - Bacteriemia - Aortic insufficiency without stenosis - Prohibitive annular size - No aortic access - Intracardiac mass or thrombus - Mobile aortic atheroma - Inability to be anticoagulated - Lack of TAVI Team or Hybrid OR - Unicuspidal and some of bicuspid types valves - Resource demanding procedure (costly)

**Table 8:** Decision Making Algorithm. Bio-AVR for older patients.

Supported by EACTS/ESC Guidelines from 2021 [6]	Supported by ACC/AHA Guidelines from 2020 [5]
- Class I SAVR for: patient desire, risks for anticoagulation, life expectancy lower than the presumed durability of the prothesis, previous mechanical valve thrombosis, STS-PROM/ EuroSCORE II < 4% and operable but unsuitable for TAVI - Class IIa for low likelihood, low risks for future redo or aged >65 years - Class IIb for patients already on NOAC for high risks of thromboembolism.	- Class 1 SAVR for symptomatic, 65–80 years old, shared decision for the type of valve/approach: SAVR or TF-TAVI - Class 1 SAVR for asymptomatic well-informed patients of any age who ask for this type of valve, with contraindication for anticoagulation, with or without depressed LVEF (<50%), >20 years of life expectancy, rapid progression or urgency (abnormal exercise test, elevated BNP) or unsuitable for TF-TAVI - Class 2a for >65 years - Class 2b percutaneous balloon dilatation as bridge to SAVR or TAVI for critically ill patient
**Pros**	**Cons**
- Most of the previously mentioned advantages - Lowest rate of degeneration - Alternative access available - Minimally invasive techniques associated with Rapid Deployment Valves can have an interest - Aortic root enlargement is possible in order to prepare for a later TAVI	- Nowadays the full sternotomy may be considered a bit invasive - More BAV with Perceval Valve - Minimally invasive techniques are not accessible in all centers

**Table 9:** Decision Making Algorithm. Valve-in-Valve.

Not mentioned by guidelines	
Pros	Cons
- Ultimate alternative for inoperable patients with biological prothesis already in aortic position.	- Low coronary ostia - Narrow sinuses of Valsalva - Tall leaflets - High supra-annular position - High risk of PPM

For patients over 65, the development of "valve in valve" TAVI techniques makes the choice of a bio prostheses even more logical. In fact, it avoids reoperation at an advanced age with a higher risk [63].

The whole point of the first surgery will then be to enable and prepare the implantation of a TAVI later (enlargement of the annulus and replacement of the aorta in case of small anatomy, radiopaque prostheses, Bentall, although at higher risk, to avoid coronary sequestration etc).

To date, there is no perfect substitute, and we are forced to put in balance our decisions, adapting our strategies in function of multiples parameters.

At a closer look to the actual’s guidelines, we observe that most of the recommendation are between of what semantically define the younger, middle and older age. The trespassing of those conventional borders offers to the Heart Teams an important role in choosing the right approach. With the life expectancy growing, we are witnessing to an increasing category of patients, around 80 years old, who are largely covered by THVs.

A full range of options are becoming available for the patients of 50–70 years old. All around the biological age, the key factor seems to be their physical age with life expectancy and comorbidities.

The question regarding the choice of substitute is of particular importance in young patients under 65 and especially under 50.

In fact, young patients present a more rapid deterioration of biological prostheses (SVD) and due to their active life are also more often concerned with the possible risks of anticoagulant treatments for mechanical prostheses.

The ROSS procedure with the placement of an aortic pulmonary autograft is still underused [36,64] This technique has shown very good results in young and middle-aged patients [37] and it is more interesting in young patients who have a contraindication to anticoagulants. As it is a technically challenging operation, it involves a dedicated team in high volume centers. In all cases this is the choice of the patient after proposition of the ideal substitute by the heart team.

*In this category of age, for extensive endocarditis, another possible solution is the use of homografts. They proved their utility and have good hemodynamics. The Achille heel of this technique is the calcic degeneration with very complex reintervention dues to extensive adherences. To date, they are not mentioned by the European or Americans Guidelines.

**Although rare at younger age, TAVI is a Class 1 of recommendation for high prohibitive surgical risk with >1 year of life expectancy **(5).**

## 2. Special Considerations and Future Directions

Bearing in mind that most of our therapeutic decisions rely on actual American and European Guidelines, one must consider a series of morpho-pathologies which require particular discussion. Thus, for the younger population, bicuspid valves, small aortic annulus and patients with intermediate ascending aorta dilatation are of particular interest. As far as TAVI is concerned, for the older population, difficult access, low coronary height, heavy annular and LVOT calcification are among the most common concerns. Furthermore, apart from inherited morphologies, they evince age related acquired pathologies.

Thus, with regard to the younger population, it has been proved that valve phenotype is not a contraindication in the case of patients taken into consideration for the Ross procedure [57,65]. As previously mentioned, the ideal anatomy for this operation is the normal aortic annulus. The technique described by Williams [50] and used also by the group of Montreal consists in a lower cutoff for the ascending aorta diameter and replacement with a Dacron conduit as a stabilization method. In expert centers, with one procedure it may be possible to correct a complex pathology. Other technical additions to further increase durability through the stabilization of the homograft [39].

On the other hand, when the Ross procedure is not an option and/or we face a small aortic annulus, in addition to the annular enlargement techniques described above one of the possibilities is an oversized supra-annular mechanical prothesis. For the middle-aged population, Gillinov et al. reported a five-year survival of 74%, and freedom from thromboembolism and hemorrhage was of 90% [66].

Roedler et al. reported that five- and ten-year survival was of 83% and 67% respectively. Over the follow-up, the mean gradient for the group receiving a 21 mm prothesis was between 23.4 and 25.3 mmHg [67].

Still another situation to avoid is the one related to PPM as a strong and independent predictor of short-term mortality among patients undergoing AVR, and its impact is related both to its degree of severity and the status of left ventricular function. In contrast to other risk factors, moderate-severe PPM can be largely avoided with the use of a prospective strategy at the time of operation [68]. A tool for predicting PPM after SAVR was proposed by Pibarot [69]: multiplying the patient’s BSA by 0.85 calculates the minimum EOA value required to prevent PPM. EOA with aortic root enlargement, may offer the solution which would allow surgeons to implant the appropriate prosthesis in young and middle aged patients.

Usually seen as the option reserved for the elderly population, TAVI has also several contraindications.

Patient with unicuspid, same type of bicuspid valves, heavily calcified LVOT (<2 cm) or low coronary high (<1 cm) are not suitable for TAVI. For this type of scenario, mini invasive surgery with or without rapid deployment valves are preferable.

The International Expert Consensus on Sutureless and Rapid Deployment Valves in Aortic Valve Replacement Using Minimally Invasive Approaches from 2016 adopted a series of 12 recommendations [70].

Their first statement refers to the use of sutureless and rapid deployment valves together with minimally invasive approaches in patients requiring biological valve replacement and not serving as candidates for TAVI. They found annular sizes of 19 to 27 mm suitable for these types of approaches and implantation is also possible in bicuspid valves [70]. Despite limited supportive data [71,72], a surgical AVR as first approach followed by a TAVI valve-in-valve intervention in case of valve failure, has been proposed to younger patients. To date, most of the existing studies have been performed with commonly available valves [3,4]; however, not with the newest ones.

In light of the aforementioned facts, the Inspiris Resilia aortic valve® (INSPIRIS) (Edwards Lifesciences LLC, Irvine, CA, USA) is a new bovine pericardial prosthesis, designed to reduce calcification and increase durability. The valve’s cobalt-chromium alloy band enables a controlled expansion to fit a new transcatheter valve (Valve-in-Valve procedures) within the existing one [73]. As the prothesis started to be implanted in 2017, the few available studies report good hemodynamics and no structural deterioration [73,74].

Overall, the marketing of the stent extendable characteristics triggers increased interest in these valves, and even if there are no reports sustaining these features, the surgical community was charmed by the concept and the valve now enjoys worldwide use.

However, reoperation because of bioprosthetic degeneration is an issue, and Valve-in-Valve therapies have gained increased acceptance. Radiolucency of stentless bioprosthetic valves, represent a significant challenge [75].

The Cor-Knot device (Cor-Knot device, LSI Solutions, Inc., Victor, NY, USA) might serve as an ideal radiopaque marker of the annular plane. The knot-tying device, consists of radiopaque Titan clips for anchoring the prosthetic valves. As several groups reported complications related to leaflets perforation, it is necessary to pay attention to optimal angulation of Cor- Knot fasteners away from the centerline of flow and supraannular rather than intraannular positioning of the aortic prosthesis remain important in avoiding problems [76]. Performance of a magnetic resonance image (MRI) is not prohibited under standard testing conditions. The undoubtable benefits for V-in-V procedures, shortening pump time and access for minimally invasive surgeries have to be validated by larger high-quality trials and studies [77,78,79]. The use of this device was associated with increased cost and consequently, the larger use is still limited.

In order to avoid anticoagulation for young patients (40–60 years), Pasala et al. proposed a paradigm shift from the current model by introducing TAVR as the first option [80]. Their supposition is that TAVR may last approximately 10 years until patients are 60 to 80 years of age, when they could undergo open heart surgery. If bioprosthetic valve dysfunction subsequently occurs when the patient is aged > 80 years, ViV TAVR can be performed [80]. Merely in order to avoid anticoagulation, various possible disadvantages are not presented in their article. Delaying surgical time by a TAVI for different types of scenarios (patient’s wishes, other comorbidities with uncertain evolution, etc.) was shown to be not ideal in a study by Jawitz [81]. SAVR following TAVR was associated with worse than expected outcomes as compared to similar patients initially undergoing SAVR. The differences were substantial in their Low-Risk group with an observed mortality 5.5 higher than the expected mortality. In selected cases, TAVR-in-TAVR seems to be a valid alternative to SAVR in degenerated transcatheter valves [82,83].

We will resume our discussion by mentioning alternative accesses for TAVI. Patient anatomy and comorbidities often determine eligibility for alternative pathways. Even if actual tendencies are to migrate to catheters, most of those techniques are surgical. In order to secure the act, those approaches should be managed by all surgeons involved.

In their paper, Overtchouk et al. offer a broad overview of those techniques [84]. Existing data does not allow TAVI operators to favor one access over another—between transcarotid, trans-subclavian and transaortic—because all have their specific strengths and weaknesses. The transapical access is progressively being abandoned as a result of its invasiveness and poor outcomes. These drawbacks encouraged operators to master other less morbid alternative pathways [84,85,86].

In the recently published European Guidelines, non-transfemoral TAVI may be considered for inoperable or unsuitable for TF-TAVI patients, being classed as a IIb recommendation [6].

## 3. Conclusions

To conclude, we might affirm that the sequence of approaches should follow the present Guidelines. Starting out from this consideration, individualization of the treatment should be the next step and represents the key to treatment success. Unfortunately, nowadays we are struggling to demystify the Ross procedure and mini-thoracotomy approaches which, even 20 years after the first report, are still considered to be extravagant procedures.

In addition to this, mention should be made of the fact that “new approaches” are not as new as they are thought to be. They are still performed mostly in high volume centers, and even if they are secure and reproductible, unfortunately, they never make it too far outside such centers.

Furthermore, due to the technical complexity, not all patients are ideal candidates and therefore surgeons must be particularly selective early on in their experience. A long-term strategy must be anticipated at the moment of initial surgery and the choice of prosthetic conduit or approach must be guided by the patient’s future needs.

Consequently, young surgeons must be supported by proctoring programs and quality debates inside Heart Teams. We hold that THVs have to be seen as complementary to surgery, and should be taught early on in surgical training. Thus, joining aortic valve team s will prove helpful in establishing a TAVI expertise, and can also introduce one to SAVR patients.

Last but not least, the real challenge lies in the management of these techniques, as well as in integrating new available materials and data from the latest studies. Overall, we can state that surgery of the aortic valve is still an ongoing process and we must continue sharing our experiences.

## Acronyms

AVR	aortic valve replacement
Bio-AVR	Bioprosthetic aortic valve replacement
CPB	Cardiopulmonary bypass
SAVR	surgical aortic valve replacement
TAVI	transcatheter aortic valve implantation
TF-TAVI	transfemoral-transcatheter aortic valve implantation
TAVR	transcatheter aortic valve replacement
RAMT	right anterior mini-thoracotomy
RALT	right anterolateral mini-thoracotomy
V-in-V	valve in valve
SVD	structural valve deterioration
PPM	patient prosthesis mismatch
EOA	effective orifice area
LVOT	left ventricle outflow tract
RDVs	Rapid Deployment Valves
THVs	Transcatheter Valves
MRI	Magnetic Resonance Image
m-AVR	Mechanical aortic valve replacement
